# Accuracy of ultrasound detecting splenic and hepatic round cell neoplasia in dogs compared to cytology and histopathology

**DOI:** 10.18849/ve.v10i4.724

**Published:** 2025-11-10

**Authors:** Ernest Martinez Martinez+

**Keywords:** CANINE, CANCER, CYTOLOGY, DIAGNOSTIC IMAGING, ROUND CELL TUMOURS, ULTRASOUND

## Abstract

**PICO question:**

In dogs, how accurate is abdominal ultrasound in detecting round cell neoplasia in the liver and spleen when compared to cytological or histopathological diagnosis?

**Clinical bottom line:**

**Category of research:**

Diagnosis.

**Number and type of study designs reviewed:**

Twelve studies were appraised in total. Ten of them were retrospective cohort studies, and only two were prospective studies.

**Strength of evidence:**

Moderate.

**Outcomes reported:**

Ultrasonography is useful for initial evaluation of canine liver and spleen but shows limited diagnostic certainty on its own. Across the studies, distinguishing benign from malignant change and differentiating among diffuse hepatopathies was inconsistent, and false negatives occurred despite normal-appearing organs. Detection of splenic involvement with aggressive mast cell disease was particularly unreliable, and ultrasound assessment of hepatic lymphoma was imperfect with overlapping appearances. Definitive case classification in the evidence base depended on cytology or histopathology rather than ultrasound alone.

**Conclusion:**

Histopathological and cytological examination remains the gold standard for diagnosis, as ultrasound on its own is an unreliable tool for diagnosis of round cell tumours involving the spleen and the liver.

## How to apply this evidence in practice

The application of evidence into practice should take into account multiple factors, not limited to: individual clinical expertise, patient’s circumstances and owners’ values, country, location or clinic where you work, the individual case in front of you, the availability of therapies and resources.

Knowledge Summaries are a resource to help reinforce or inform decision-making. They do not override the responsibility or judgement of the practitioner to do what is best for the animal in their care.

## The evidence

The strength of evidence across the studies, which involve dogs and address splenic and hepatic round cell neoplasia, varies due to differences in design and methodology. The majority of the studies are retrospective (10), which generally provide lower-quality evidence because of biases and limited control over data, highlighting ultrasound's variable sensitivity and specificity but lacking broader applicability (Book et al., 2011; Sato & Solano, 2004; Stefanello et al., 2009; Warren-Smith et al., 2012; Whiteley et al., 1989; Wrigley et al., 1988; Feeney et al., 2008; Finora et al., 2006; Crnogaj et al., 2021; Sumping et al., 2022). The two prospective studies (Crabtree et al., 2010; Ohlerth et al., 2008) offer stronger evidence by controlling data collection more effectively, yet their impact is still limited by sample size. Collectively, these studies emphasise that while ultrasound can provide useful diagnostic insights, its limitations in sensitivity and specificity necessitate complementary diagnostic methods like cytology or histology. The evidence suggests a cautious approach to changing clinical practice from diagnosis based on ultrasound alone, advocating for further prospective research to validate and refine these findings.

## Summary of the evidence

**Book et al. (2011):** Correlation of ultrasound findings, liver and spleen cytology, and prognosis in the clinical staging of high metastatic risk canine mast cell tumors **Aim: **To compare abdominal ultrasonography with cytology for detecting hepatic and splenic mast cell infiltration and to assess whether cytological findings inform prognosis.

Population:	Dogs with clinically aggressive grade II or III mast cell tumours. Location: Washington State University Veterinary Teaching Hospital, Pullman, USA.
Sample size:	19 dogs.
Intervention details:	Abdominal ultrasonography was used to assess liver and spleen. Cytologic evaluation of liver and/or spleen aspirates was performed in all the patients (19), with 10 being classified as grade II and 9 as grade III.
Study design:	Retrospective study.
Outcome Studied:	Objective assessment of the specificity and sensitivity of ultrasound for detecting mast cell infiltration and defining the prognostic role of cytologic evaluation of liver and spleen aspirates.
Main Findings (relevant to PICO question):	Sensitivity of ultrasound for detecting mast cell infiltration was 43% for the spleen (3/7) and 0% for the liver (0/3). Specificity of ultrasound abnormalities for predicting mast cell infiltration was 75% for the spleen (9/12) and 91% for the liver (10/11). The positive predictive value for detection of mast cell infiltration was 50% for the spleen (3/6) and 0% for the liver (0/1). The negative predictive value was 69% for the spleen (9/13) and 77% for the liver (10/13). Percentages above are derived from cytology-confirmed counts; x/y are shown for transparency.
Limitations:	There was a lack of histological confirmation for most cases, because cytology was used as the reference standard. The sample size was small, which reduces the precision of the accuracy estimates. This was a single-centre study, which may limit generalisability. Not all dogs had diagnostic liver aspirates, which introduces potential verification bias. Ultrasound interpretations may have been operator-dependent, which could introduce review bias.

**Crabtree et al. (2010):** Diagnostic accuracy of gray-scale ultrasonography for the detection of hepatic and splenic lymphoma in dogs **Aim: **To determine the diagnostic accuracy of greyscale ultrasonography for identifying hepatic and splenic lymphoma using cytology as the reference standard.

Population:	Dogs with a confirmed diagnosis of multicentric lymphoma. Location: Auburn University College of Veterinary Medicine, Auburn, USA.
Sample size:	28 dogs.
Intervention details:	Abdominal ultrasonography was used to assess liver and spleen. Fine-needle aspiration (FNA) samples were obtained from three sites each in both liver and spleen. Cytologic evaluation of the liver and/or spleen aspirates was performed.
Study design:	Prospective study.
Outcome Studied:	Objective assessment of the accuracy of greyscale ultrasonography in detecting hepatic and splenic lymphoma compared to cytology.
Main Findings (relevant to PICO question):	Liver (n = 69; true positives (TP) = 24, false negatives (FN) = 9, false positives (FP) = 7, true negatives (TN) = 29): sensitivity 72.7% (24/33); specificity 80.6% (29/36); positive predictive value (PPV) 77.4% (24/31); negative predictive value (NPV) 76.3% (29/38); accuracy 76.8% (53/69). Spleen (n = 72; true positives (TP) = 42, false negatives (FN) = 0, false positives (FP) = 23, true negatives (TN) = 7): sensitivity 100% (42/42); specificity 23.3% (7/30); positive predictive value (PPV) 64.6% (42/65); negative predictive value (NPV) 100% (7/7); accuracy 68.1% (49/72). Sampling adequacy: hepatic cytology 82.1% (69/84) diagnostic; splenic cytology 85.7% (72/84) diagnostic. Ultrasonographic patterns: in the liver, lymphoma was identified across all reported appearances including normal parenchyma; in the spleen, a “moth eaten” appearance had PPV 100% (10/10 evaluable), and ultrasonographically normal spleens had NPV 100% (7/7). Complications: minor complications occurred in 3/30 (10%) sampled patients (two small subcutaneous haematomas; one small volume of free peritoneal fluid); no severe events and all resolved without treatment.
Limitations:	The small sample size may affect the generalisability of the findings. Only newly diagnosed, untreated dogs were included, which may limit applicability to pretreated populations. The diagnostic sampling rate was 82.1% (69/84).

**Crnogaj et al. (2021):** The clinical correlation of findings obtained by fine needle fenestration biopsy of the canine spleen with visible ultrasound changes **Aim: **To evaluate how cytological results from splenic fine-needle fenestration biopsy relate to ultrasonographic lesion characteristics in dogs.

Population:	Dogs with visible ultrasonographic changes. Location: Faculty of Veterinary Medicine, University of Zagreb, Zagreb, Croatia.
Sample size:	130 dogs.
Intervention details:	Fine-needle fenestration biopsy (FNFB) (non-aspiration technique) of the spleen in were performed in 130 dogs, with only 75 meeting the inclusion criteria. As our PICO focuses on dogs with ultrasonographic splenic changes undergoing FNFB, we do not follow up the results from the remaining 55/130 (42.3%) dogs that did not meet these criteria.
Study design:	Retrospective study.
Outcome Studied:	To assess objectively, in dogs with ultrasonographically visible splenic changes, whether splenic FNFB cytology was clinically relevant or not, and to assess association between specific ultrasound features and those cytology categories.
Main Findings (relevant to PICO question):	Incidence of clinically relevant diagnoses was 37.3% (28/75) with 82.1% (23/28) of malignant diagnoses (42.9% (12/28) lymphoma). No correlation was found between ultrasound lesions examined and the cytological diagnoses, except for splenic heterogeneity (patchy echotexture). Lesion size > 1.74 cm on ultrasound predicted clinically relevant cytology, having a sensitivity of 91.3% and specificity of 42.1% for predicting clinically relevant findings.
Limitations:	The procedures were conducted by different practitioners, therefore results will have operator variability in both ultrasonographic assessment and sampling. Retrospective design with inherent limitations in data collection and image consistency. There was lack of histological evaluation. The clinically relevant group was not limited to round cell neoplasia.

**Feeney et al. (2008):** Statistical relevance of ultrasonographic criteria in the assessment of diffuse liver disease in dogs and cats **Aim: **To test whether specific ultrasonographic criteria can discriminate among categories of diffuse hepatic disease, including round-cell neoplasia, in dogs and cats.

Population:	Dogs and cats with diffuse liver disease, evaluated using ultrasonography and confirmed with histology or cytology. Location: University of Minnesota Veterinary Medical Center, Saint Paul, USA.
Sample size:	333 patients (229 dogs and 104 cats).
Intervention details:	Retrospective and independent analysis of ultrasonographic images by three radiologists. There were eight histologic or cytologic categories: normal, inflammation, round cell neoplasia, non-round-cell infiltrative, pre nodular (early) metastatic neoplasia, lipidosis, vacuolar hepatopathy, and other. Applied ultrasonographic criteria included liver echogenicity, echotexture, borders, and edges; portal venous clarity; sound attenuation of liver parenchyma; gallbladder wall thickness and content; bile duct diameter; caudal vena cava status; and hepatic vein diameter.
Study design:	Retrospective study.
Outcome Studied:	Accuracy of ultrasonographic criteria in distinguishing eight defined categories of diffuse liver disease in dogs and cats.
Main Findings (relevant to PICO question):	Ultrasonographic appearance was insufficient to discriminate among canine and feline diffuse infiltrative liver disease, including round cell neoplasia.
Limitations:	The retrospective design has inherent limitations in data collection and image consistency. The study was limited to diffuse changes, excluding the cases with nodular or focal hepatopathy. Histologic or necropsic evaluation was not available in every case. The subjective ultrasonographic criteria limits the reproducibility of the study.

**Finora et al. (2006):** Cytological comparison of fine-needle aspirates of liver and spleen of normal dogs and of dogs with cutaneous mast cell tumours and an ultrasonographically normal appearing liver and spleen **Aim: **To compare hepatic and splenic cytology between unaffected dogs and dogs with cutaneous mast cell tumours but ultrasonographically normal organs, to judge the value of routine staging aspirates.

Population:	Dogs with cutaneous mast cell tumours (cMCT) and ultrasonographically normal liver and spleen, and clinically normal dogs. Location: Animal Medical Center, New York, USA; and College of Veterinary Medicine, Colorado State University, Fort Collins, Colorado, USA.
Sample size:	83 dogs.
Intervention details:	The cohort comprised 51 affected dogs (23 grade II and 28 grade III) and 32 clinically normal dogs. Fine-needle aspiration (FNA) of the liver and spleen were performed in both groups. The cytology findings were compared between affected and unaffected dogs.
Study design:	Retrospective study.
Outcome Studied:	Evaluation of the utility of routine hepatic and splenic aspirates in the staging of cMCT.
Main Findings (relevant to PICO question):	There was no significant difference in liver aspirates between affected and unaffected dogs. The affected dogs had significantly more mast cells per cluster (P = 0.04) and more isolated mast cells per slide (P = 0.03) in the spleen. There was considerable overlap in mast cell numbers between both patient groups (normal and dogs with cMCT), making differentiation unreliable (i.e., many indivicual cMCT dogs had counts within the range observed in normal dogs and vice versa). This overlap indicates that routine aspiration of an ultrasonographically normal liver and spleen in cMCT staging is not clinically useful.
Limitations:	The retrospective design limits control over inclusion criteria and data capture. Histopathology of liver and/or spleen was not performed in affected dogs, limiting confirmation of cytology. Subjective interpretation of mast cell numbers in cytology and absence of clear cutoff values for distinguishing normal from neoplastic mast cell infiltration. This may impair reproducibility.

**Ohlerth et al. (2008):** Contrast Harmonic Imaging Characterization of Canine Splenic Lesions **Aim: **To determine whether contrast harmonic ultrasonography can distinguish benign from malignant splenic lesions relative to cytology or histology.

Population:	Dogs with splenic abnormalities detected during abdominal ultrasonography. Location: Vertsuisse Faculty, University of Zurich, Switzerland.
Sample size:	60 dogs.
Intervention details:	Contrast harmonic ultrasonography was used after splenic abnormalities were detected during routine B-mode abdominal ultrasonography. The diagnosis was based on ultrasound-guided aspirates or histopathologic samples taken during surgery or necropsy.
Study design:	Prospective study.
Outcome Studied:	Objective assessment of the ability of contrast harmonic imaging to differentiate benign from malignant splenic lesions.
Main Findings (relevant to PICO question):	The presence of marked enhancement is of limited value as it occurred in both benign and malignant splenic lesions. Therefore, fine needle aspiration or tissue core biopsy is needed. Extensive to moderate hypoechogenicity of a splenic lesion during all blood pool phases was highly associated with malignancy.
Limitations:	Statistical analysis included different types of splenic tumours, not only round cell neoplasia – possible bias during the evaluation of subjective contrast harmonic ultrasound criteria. Only cytology was performed in most of the investigated lesions. Histology or cytology of the normal splenic tissue of diseased spleen was not performed.

**Sato & Solano (2004):** Ultrasonographic findings in abdominal mast cell disease: a retrospective study of 19 patients **Aim: **To describe the ultrasonographic features associated with confirmed mast cell infiltration in dogs and cats.

Population:	Survey of ultrasound records in dogs and cats with cytologically or histopathologically confirmed abdominal cell disease.
Sample size:	19 patients (12 dogs and 7 cats).
Intervention details:	For the purpose of this Knowledge Summary, the dog's findings only will be followed up on. Ultrasound findings were correlated with confirmed mast cell infiltration in the Final diagnoses were obtained by fine-needle aspiration, ultrasound-guided core biopsy of the affected organs, laparotomy, or necropsy.
Study design:	Retrospective study.
Outcome Studied:	Ultrasonographic features of hepatic, splenic, lymphatic, gastrointestinal, and renal mast cell infiltration.
Main Findings (relevant to PICO question):	Ultrasonographic findings in dogs with mast cell infiltration included increased liver size, a diffuse increase in liver echogenicity, and presence of one or more hypoechoic nodules in the liver or spleen. Mast cell infiltration was found in 2 ultrasonographically unremarkable livers and one spleen. Hence, a normal ultrasonographic appearance of the liver or spleen does not rule out mast cell infiltration, highlighting the importance of cytology or histopathology.
Limitations:	The small cohort reduces precision and external validity. The retrospective design limits control over imaging protocols, data quality and consistency. The study was carried out over 20 years ago and the findings are based on ultrasound equipment at the time, which limits comparability with current practice.

**Stefanello et al. (2009):** Ultrasound-Guided Cytology of Spleen and Liver: A Prognostic Tool in Canine Cutaneous Mast Cell Tumor **Aim: **To assess whether cytological evidence of hepatic or splenic mast cell infiltration predicts survival in dogs with cutaneous mast cell tumours.

Population:	Dogs with cutaneous mast cell tumours (cMCT), with and without evidence of mast cell infiltration in the spleen and liver. Location: University of Milan, Milan, Italy.
Sample size:	52 dogs.
Intervention details:	10 dogs with mast cell infiltration and 42 without. Ultrasound-guided cytology of spleen and liver was performed. The cytology findings and survival time were compared in dogs that had positive and negative results.
Study design:	Retrospective study.
Outcome Studied:	Survival times of dogs with and without cytological evidence of mast cell infiltration in the spleen and liver.
Main Findings (relevant to PICO question):	19% of dogs (10/52) had cytological evidence of mast cell infiltration in spleen or liver. Dogs with positive cytological evidence of mast cell infiltration had significantly shorter survival times (median 34 days vs 733 days, P = 0.0001). 70% (7/10) of the dogs with mast cell infiltration had two or more ultrasonographic abnormalities in the spleen and/or liver. Cytology of spleen and liver is recommended in staging, regardless of the ultrasonographic appearance.
Limitations:	The retrospective design of the study limits the statistical power and external validity. The absence of histopathological confirmation for cytologic mast cell infiltration may allow misclassification. The small sample size in the group with mast cell infiltration.

**Sumping et al. (2022):** Diagnostic accuracy of ultrasonography to detect hepatic and splenic lymphomatous infiltration in dogs and cats **Aim: **To quantify the accuracy of ultrasonography for detecting hepatic and splenic lymphomatous infiltration and to identify sonographic features associated with infiltration or immunophenotype.

Population:	Dogs and cats with cytologically or histologically confirmed lymphoma. Location: Small Animal Teaching Hospital, University of Liverpool, UK.
Sample size:	161 animals (132 dogs and 29 cats).
Intervention details:	Blinded evaluation of ultrasonographic images of the liver and spleen were taken contemporaneously with the diagnosis by two board-certified veterinary radiologists. The diagnosis of lymphoma was confirmed on cytology and/or histopathology.
Study design:	Blinded retrospective evaluation.
Outcome Studied:	One aim was to determine the diagnostic accuracy of ultrasonography in the detection of lymphomatous infiltration of the liver and spleen in a population of dogs and cats with lymphoma. Another aim was to determine if specific ultrasonographic features of the liver and spleen in dogs are associated with lymphomatous infiltration or a specific immunophenotype of multicentric lymphoma.
Main Findings (relevant to PICO question):	For the liver: sensitivity 15.8% (true positives (TP) 9 / [TP+false negatives (FN) 57] = 9/57); specificity 91.1% (true negatives (TN) 41 / [TN+false positives (FP) 45] = 41/45); positive predictive value (PPV) 69.2% (TP/(TP+FP) = 9/13); negative predictive value (NPV) 46.1% (TN/(TN+FN) = 41/89); accuracy 49.0% ((TP+TN)/total = 50/102). For the spleen: sensitivity 77.5% (TP 55 / [TP+FN 71] = 55/71); specificity 91.5% (TN 43 / [TN+FP 47] = 43/47); PPV 93.2% (TP/(TP+FP) = 55/59); NPV 72.9% (TN/(TN+FN) = 43/59); accuracy 83.1% ((TP+TN)/total = 98/118). Hence, ultrasonography of the spleen and liver is specific but not sensitive in the detection of lymphomatous infiltration. In dogs, an ultrasonographically normal liver was associated with not having lymphomatous A leopard-spotted splenic parenchyma in dogs is highly specific for lymphomatous infiltration and predicted a specific immunophenotype of multicentric lymphoma.
Limitations:	The retrospective design and review of still images limit standardisation and may introduce selection and information bias. Multiple operators and different ultrasound machines/probes may increase variability. Both radiologists were trained at the same institution, which may have introduced bias in the interpretation of images and interobserver agreement. Using cytology and histology as mixed reference standards may introduce verification bias.

**Warren-Smith et al. (2012):** Lack of associations between ultrasonographic appearance of parenchymal lesions of the canine liver and histological diagnosis **Aim: **To determine whether ultrasonographic features of canine liver lesions are associated with histological diagnoses.

Population:	Dogs that had abdominal ultrasonography and abnormal liver findings on biopsy or necropsy. Location: Queen Mother Hospital for Animals, Royal Veterinary College, North Mymms, UK.
Sample size:	371 dogs.
Intervention details:	Abdominal ultrasound followed by biopsy or necropsy for histological diagnosis.
Study design:	Retrospective review.
Outcome Studied:	Correlating ultrasonographic features and histological analysis of abnormal liver.
Main Findings (relevant to PICO question):	No statistically significant associations were found between specific ultrasonographic features and histological diagnoses. Ultrasonography is an insensitive test and tentative diagnoses should not be performed based on ultrasonographic findings alone. Histological examination remains essential for accurate diagnosis of liver disease.
Limitations:	The retrospective design with multiple observers may introduce variability and lesion-matching imprecision. The focus on the liver limits the ability to generalise to other abdominal organs. The differences between imaging and sampling locations may have affected concordance.

**Whiteley et al. (1989):** Ultrasonographic appearance of primary and metastatic canine hepatic tumors. A review of 48 cases **Aim: **To characterise ultrasonographic patterns of primary and metastatic hepatic tumours in dogs and relate them to tumour types.

Population:	Dogs with primary and metastatic hepatic neoplasia. Location: University of Minnesota, Veterinary Teaching Hospital, Saint Paul, USA.
Sample size:	48 dogs.
Intervention details:	Ultrasonographic findings in dogs with histological confirmation. The analysis was focused on the correlation between sonographic appearance and neoplastic cell types.
Study design:	Retrospective study.
Outcome Studied:	Ultrasonographic patterns associated with different types of hepatic neoplasms, including primary and metastatic tumours.
Main Findings (relevant to PICO question):	Focal hyperechoic masses were frequently hepatocellular carcinomas (14/15 dogs). Two patterns were noted for lymphosarcoma: diffuse mildly hyperechoic (6/11) and multifocal hypoechoic (5/11). No specific sonographic pattern could reliably predict the neoplastic cell type for focal or multifocal hypoechoic lesions. Subtle sonographic changes in the presence of elevated liver enzymes should prompt a biopsy to distinguish between neoplastic and non-neoplastic processes.
Limitations:	Retrospective design and small numbers with tumour subtypes limit statistical analysis and external validity. The study was carried out over 20 years ago and the findings are based on ultrasound equipment at the time, which limits comparability with current practice.

**Wrigley et al. (1988):** Ultrasonographic features of splenic lymphosarcoma in dogs: 12 cases (1980-1986) **Aim: **To describe the ultrasonographic characteristics of splenic lymphosarcoma in dogs and their usefulness for guiding cytologic or histologic confirmation.

Population:	Dogs with histologically confirmed splenic lymphosarcoma. Location: Colorado State University, Fort Collins, USA.
Sample size:	12 dogs.
Intervention details:	Ultrasonographic features in dogs diagnosed with splenic lymphosarcoma were reviewed. The spleen and adjacent organs were assessed.
Study design:	Retrospective study.
Outcome Studied:	Ultrasonographic patterns observed in splenic lymphosarcoma including nodule characteristics and spleen echogenicity.
Main Findings (relevant to PICO question):	All dogs exhibited poorly marginated hypoechoic to anechoic nodules ranging from 4 mm to 3 cm in diameter. A hypoechoic spleen was noted in 9/12 dogs when compared with the liver or renal cortices. Recognition of these patterns can be a useful diagnostic tool for splenic lymphosarcoma, and ultrasonographically guided needle aspiration will help confirming the diagnosis histologically.
Limitations:	The retrospective design limits control over image acquisition, quality and consistency. The small sample size reduces precision and external validity. The study was carried out over 20 years ago and the findings are based on ultrasound equipment at the time, which limits comparability with current practice.

## Appraisal, application and reflection

The reviewed papers provide valuable insights into the use of abdominal ultrasound for detecting round cell neoplasia (including mast cell tumours and lymphoma) in the liver and spleen of dogs. Although the studies varied in focus and methodology, they converge on several key points that are relevant to the PICO question.

The consensus amongst the studies is that ultrasonography is limited as a standalone diagnostic tool for the detection of round cell neoplasia in the liver and spleen of dogs. While it can provide useful preliminary information (for example, a honeycomb-like echotexture of the spleen is highly suggestive of lymphoma in dogs, as noted in Wrigley et al. (1988). In addition, hypoechoic nodules in the liver and spleen may indicate mast cell infiltration, as described in Sato & Solano, (2004), its limitations necessitate a multimodal approach including cytology or histopathology to reach an accurate diagnosis. For instance, Book et al. (2011) advocate for routine splenic aspiration in cases of aggressive mast cell tumours, regardless of the ultrasonographic appearance, due to the low sensitivity of ultrasound (43% (3/7) for the spleen and 0% (0/3) for the liver). This approach aligns with the conclusion from other authors, such as Crabtree et al. (2010), who suggest performing aspirates from the spleen when abnormalities are detected, and from the liver regardless of ultrasound findings when investigating canine multicentric lymphoma.

Many of the included studies are retrospective and have small sample sizes, which limits the strength of inference. To be specific by species and study: Sato & Solano (2004) reported a retrospective case series of 19 patients comprising 12 dogs and 7 cats; Book et al. (2011) reported a retrospective study of 19 dogs; and Crabtree et al. (2010) reported a structured prospective study of 28 dogs. The inconsistency in findings across the different studies also illustrates the variability in ultrasonographic interpretations, most likely related to differences in equipment and operator expertise. For example, Feeney et al. (2008) found that ultrasonographic appearance alone was insufficient to discriminate among different types of diffuse liver diseases, including round cell neoplasia, further emphasising the need for cytological or histopathological confirmation.

From an evidence-based veterinary medicine (EBVM) perspective, the limitations of the available studies should be carefully considered when applying findings to clinical practice. The hierarchy of evidence places randomised controlled trials (RCTs) and systematic reviews at the top, as they provide the most reliable data with minimal bias (Howick et al., 2011). However, the majority (10/12) of the studies in this Knowledge Summary are retrospective studies, which are inherently more susceptible to selection bias, confounding variables, and inconsistent data collection. While these studies provide valuable clinical insights, they rank lower in the evidence pyramid and should ideally be supplemented with higher-level research, such as prospective cohort studies with predefined criteria for ultrasonographic abnormalities and their correlation with cytological and histopathological findings.

Another key EBVM principle relevant to this discussion is diagnostic accuracy assessment. Measures such as sensitivity, specificity, positive predictive value (PPV), and negative predictive value (NPV) are crucial when evaluating the reliability of ultrasound as a diagnostic tool. Many of the included studies report low sensitivity, meaning a negative ultrasound does not reliably exclude disease, while specificity varies depending on tumour type and organ involvement. For example, ultrasound sensitivity for detecting mast cell infiltration was 43% (3/7) for the spleen and 0% (0/3) for the liver (Book et al., 2011). For lymphoma, ultrasound sensitivity was 72.7% (24/33) for the liver and 100% (42/42) for the spleen, but specificity was only 23.3% (7/30) for the spleen, leading to a high rate of false positives (Crabtree et al., 2010). In Sato & Solano (2004), mast cell infiltration was found in two ultrasonographically normal livers and one spleen, further highlighting the limitations of ultrasound in ruling out disease.

The potential for operator variability further complicates the interpretation of ultrasound findings, emphasising the need for standardised training and imaging protocols to improve reproducibility and reduce diagnostic uncertainty. For example, Feeney et al. (2008) noted that subjective ultrasonographic criteria (e.g., liver echogenicity, echotexture) were insufficient to reliably distinguish between different types of liver disease, including round cell neoplasia.

Future studies should ideally focus on prospective evaluations with well-defined inclusion criteria, larger sample sizes, and blinded comparisons between ultrasound findings and histopathology to establish clearer guidelines for the use of ultrasonography in detecting round cell neoplasia in dogs. For instance, Sumping et al. (2022) demonstrated that ultrasonography had a high specificity (91.0% for the liver and 93.9% for the spleen) but low sensitivity (16.7% for the liver and 73.1% for the spleen) in detecting lymphomatous infiltration, further supporting the need for cytological or histopathological confirmation.

Thus, while ultrasonography remains a key tool in screening dogs for round cell infiltration, concomitant cytological or histological examination ensures a more reliable and comprehensive diagnostic approach. This is particularly important in cases of aggressive mast cell tumours or lymphoma, where early and accurate diagnosis can significantly impact prognosis and treatment outcomes.

## Methodology

**Search Strategy:** 

Databases searched and dates covered:	CAB Abstracts on OVID Platform (1973–Week 9 2025) PubMed accessed via the NCBI website (1920–1 March 2025)
Search strategy:	CAB Abstracts: (dog or dogs or canine or canines or bitch or bitches).mp. or exp dogs/ or exp bitches/ ((liver* or hepatic* or spleen* or splenic*).mp. or exp liver/ or exp spleen/) and (round cell neoplas* or round cell tumour* or round cell tumor* or lymphoma* or lymphosarcoma* or mast cell tumour* or mast cell tumor* or MCT or histiocytic sarcoma or plasma cell tumour or plasma cell tumor or multiple myeloma).mp. (ultrasound or ultrasonograph* or sonograph*).mp. or exp ultrasonography/ or exp ultrasound/ or exp ultrasonic diagnosis/ (cytolog* or histolog* or histopatholog* or biopsy).mp. or exp cytology/ or exp histopathology/ 1 and 2 and 3 and 4 PubMed: dog OR canine (liver OR hepatic OR spleen OR splenic) AND ('round cell neoplasia' OR 'round cell tumour' OR 'round cell tumor' OR lymphoma OR lymphosarcoma OR 'mast cell tumour' OR 'mast cell tumor' OR MCT OR ‘histiocytic sarcoma’ OR ‘plasma cell tumour’ OR ‘plasma cell tumor’ OR ‘multiple myeloma’) ultrasound OR sonography OR ultrasonography histology OR histopathology OR biopsy OR cytology 1 AND 2 AND 3 AND 4
Dates searches performed:	1 Mar 2025

**Exclusion / Inclusion Criteria:** 

Exclusion:	Does not answer the PICO question, non-English language, popular press (non-scientific or peer-reviewed), human medicine literature.
Inclusion:	Any primary veterinary research/systematic review that compared the accuracy of ultrasound compared to cytology / histopathology.

**Search Outcome:** 

Database	Number of results	Excluded – did not answer the PICO question	Excluded – conference abstracts or popular press	Excluded – not accessible	Total relevant papers
CAB Abstracts	117	98	9	0	10
PubMed	86	73	2	0	11
Total relevant papers when duplicates removed					12

## ORCiD

Ernest Martinez Martinez: https://orcid.org/0009-0003-8592-3989

## Conflict of Interest

The author declares no conflicts of interest.
